# Gemcitabine Direct Electrochemical Detection from Pharmaceutical Formulations Using a Boron-Doped Diamond Electrode

**DOI:** 10.3390/ph14090912

**Published:** 2021-09-10

**Authors:** Iulia Rus, Alexandra Pusta, Mihaela Tertiș, Cristina Barbălată, Ioan Tomuță, Robert Săndulescu, Cecilia Cristea

**Affiliations:** 1 Department of Analytical Chemistry, Iuliu Hațieganu University of Medicine and Pharmacy, 4 Louis Pasteur Street, 400349 Cluj-Napoca, Romania; rus.iulia@umfcluj.ro (I.R.); alexandrapusta@gmail.com (A.P.); mihaela.tertis@umfcluj.ro (M.T.); rsandulescu@umfcluj.ro (R.S.); 2 Department of Medical Devices, Iuliu Hațieganu University of Medicine and Pharmacy, 4 Louis Pasteur Street, 400349 Cluj-Napoca, Romania; 3 Department of Pharmaceutical Technology and Biopharmaceutics, Iuliu Hațieganu University of Medicine and Pharmacy, 41 Victor Babeș Street, 400012 Cluj-Napoca, Romania; barbalata.cristina@umfcluj.ro (C.B.); tomutaioan@umfcluj.ro (I.T.)

**Keywords:** gemcitabine, boron-doped diamond electrode, differential pulse voltammetry, amperometry, pharmaceutical formulations

## Abstract

The development of fast and easy-to-use methods for gemcitabine detection is of great interest for pharmaceutical formulation control in both research laboratories and hospitals. In this study, we report a simple, fast and direct electrochemical method for gemcitabine detection using a boron-doped diamond electrode. The electrochemical oxidation of gemcitabine on a boron-doped diamond electrode was found to be irreversible in differential pulse voltammetry, and scan rate influence studies demonstrated that the process is diffusion-controlled. The influence of the pH and supporting electrolytes were also tested, and the optimized differential pulse voltammetry method was linear in the range of 2.5–50 μg/mL, with a detection limit of 0.85 μg/mL in phosphate-buffered saline (pH 7.4; 0.1 M). An amperometric method was also optimized for gemcitabine detection. The linear range of the method was 0.5–65 μg/mL in phosphate-buffered saline of pH 7.4 as well as pH 5.5, the limit of detection being 0.15 μg/mL. The optimized differential pulse voltammetry and amperometric detection strategies were successfully applied to pharmaceutical formulations, and the results were compared to those obtained by high-performance liquid chromatography and UV-Vis spectrophotometry with good correlations.

## 1. Introduction

Gemcitabine (2′,2′-difluoro 2′deoxycytidine–(GMB)) is a pyrimidine nucleoside antimetabolite drug, which can be used in the treatment of a variety of cancers, such as non-small cell lung cancer, pancreatic, breast and bladder cancer [1,2], as well as non-Hodgkin’s lymphoma [1]. GMB is a prodrug requiring activation by intracellular phosphorylation to exhibit antitumor activity [3]. Upon activation, the resulting GMB di- and triphosphate inhibit DNA synthesis and the activity of ribonucleotide reductase, thus leading to tumor cell death [3,4]. GMB is rapidly inactivated intracellularly, being converted to its metabolite 2′,2′-difluoro-deoxyuridine [4]. GMB is administered intravenously, either alone or in combination with other chemotherapeutic agents [1,2]. GMB pharmaceutical formulations include: an infusion solution, concentrate for the infusion solution or lyophilized powder for the infusion solution.

Given the extended use of GMB in therapy, it is necessary to develop analytical techniques that can be easily applied for its detection in pharmaceutical formulations.

Like most chemotherapeutic agents, GMB is dosed in patients using the body surface area [2], so that each patient receives a personalized dose. Moreover, the infusion solution is prepared ex tempore for each patient. Both of these aspects can lead to errors in the preparation or dosing process, which can result in improper concentrations in the final solution that is administered to the patient. In recent years, there has been an increase in interest for the development of new pharmaceutical formulations containing antitumor drugs. These strategies include the use of liposomes [5,6], dendrimers [7,8] or nanoparticles [9] and require the development of analytical methods for the quantification of the loading efficiency, loading capacity and release rate in physiological media and tumor media, respectively, from these carriers.

Taken all the aforementioned reasons into account, it is important to develop a fast, simple and reliable method of GMB detection from pharmaceutical samples. A versatile method that could be used both in a hospital setting for the infusion solution control and in research laboratories for the development of new pharmaceutical formulations would be of great interest.

Different types of strategies were reported for the detection of GMB, such as chromatographic methods [10,11,12,13], which are also employed by the European Pharmacopoeia 10th edition [14], as well as spectrophotometric [15] and electrochemical methods [16,17,18,19,20,21]. The latter represent a promising alternative, since they are simple, fast and require small quantities of samples. The direct electrochemical detection of GMB on a carbon paste electrode has been reported by Teradal et al. [16]. However, this method requires the addition of a surfactant, which could not be feasible for solutions that are prepared ex tempore and need to be administered to the patient after analysis. Another direct approach involved the use of gold electrodes for GMB detection [17]. The main disadvantage of this method is its need for high pH values and, therefore, the need for sample pH modification for detection. Indirect approaches included the use of GMB interactions with DNA [21] or the use of molecularly imprinted polymers (MIP) with or without DNA probes [18,19,20] for GMB electrochemical detection. Despite the very low limits of detection of some of these methods [18], the modification of the electrode surface with either DNA or MIP requires complex steps, and such surfaces can become unstable over time.

The boron-doped diamond electrode (BDDE) is an attractive option for GMB detection, since it has numerous advantages, such as a large electrochemical potential window, small and stable background current, reduced fouling compared to noble metal electrodes and great stability over time [22].

In this study, we reported for the first time a direct electrochemical method for the detection of GMB using the boron-doped diamond electrode. Two electrochemical detection strategies were optimized, one using differential pulse voltammetry (DPV) and the other one using amperometry. The voltametric method is convenient due to its short analysis time, ease of use and applicability at physiological pH 7.4. The influence of the pH was studied on a wide range of pH values using DPV. The influence of the scan rate was investigated using DPV in order to assess the electrochemical behavior of GMB. Antitumor drug detection was also carried out using amperometry in phosphate-buffered solutions with a pH similar to that of the physiological (7.4) and tumor media, respectively (5.5). Finally, both DPV and amperometric detection strategies were applied to pharmaceutical formulations, yielding good recovery rates for the pharmaceutical formulations tested. The results obtained from the GMB analysis using the developed electrochemical methods were compared to those obtained by HPLC-UV and UV-Vis spectrophotometry, confirming that there was good correspondence between all four methods. At the same time, the correlation of the methods demonstrated the accuracy and robustness of the optimized electrochemical detection strategies.

## 2. Results and Discussions

### 2.1. Electrochemical Characterization of Gemcitabine

#### 2.1.1. The Influence of the Electrode Material on the Detection of Gemcitabine

Standard GMB samples were tested on different electrode materials like glassy carbon electrode (GCE), graphite or platinum-based screen-printed electrodes (SPEs), pencil graphite electrodes (PGE) and PGE modified with gold nanoparticles (AuNPs), and no oxidation or reduction peak was observed in the range of 0–1.5 V. Despite the data available in the literature [17], no oxidation signal of GMB was observed on gold-based SPEs in the same potential range. Since the BDDE has a large potential window and it can withstand higher potential values, the oxidation signal of GMB was studied in the range of 0.5–2.5 V on BDDE, and an oxidation peak was noticed around 2.2 V in DPV (see Figure S1 from the Supplementary File).

#### 2.1.2. The Influence of the pH and Supporting Electrolyte on the Detection of Gemcitabine

**Figure 1:**
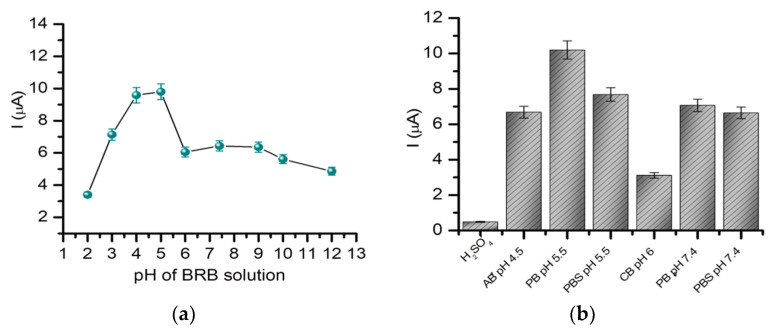
(**a**) Variation of the intensity of the oxidation signal of GMB in BRB solutions of different pH (2–12) on BDDE using DPV (scan rate: 100 mV/s, potential range: 0.5–2.5 V). (**b**) Variations of the intensity of the oxidation current with the electrolyte solution.

The oxidation signal of GMB was studied in Britton Robinson buffer (BRB) solutions on a wide range of pH, as can be observed in Figure 1. The intensity of the oxidation current increased from pH 2, reaching the highest intensity at pH 5; after which, it decreased to relatively constant values between pH 6 and 12. A similar behavior was observed in another study in which GMB detection was performed using a carbon paste electrode [16]. Different electrolytes were also tested, and the best results were obtained for the phosphate buffer (PB) (pH 5.5; 0.1 M), followed by phosphate-buffered saline (PBS) (pH 5.5; 0.1 M), PB (pH 7.4; 0.05 M), PBS (pH 7.4; 0.05 M) and acetate buffer (AB) (pH 4.5; 0.1 M), with similar values to the intensity. In the citrate buffer (CB) solution (pH 6; 0.1 M), the intensity of the signal was significantly smaller. The oxidation signal of GMB was also investigated in the carbonate buffer (CBB) (pH 10; 0.1 M), but no oxidation peak was observed between 0.5 and 2.5 V. Considering the purpose of the sensor’s development, the following tests were performed in PBS (pH 7.4; 0.05 M). A variation of the oxidation potential of GMB with the pH was also observed (see Figure S2 from the Supplementary File). The value of the potential decreased with the increase in the pH with 21.4-mV pH^−1^, as indicated by the slope value of the equation obtained by following the pH dependence of the potential. The slope was far from the expected value of 59 mV pH^−1^; therefore, we could not estimate the correlation between the number of electrons and protons involved in the electro-oxidation process [21].

#### 2.1.3. The Influence of the Scan Rate on the Detection of Gemcitabine

**Figure 2:**
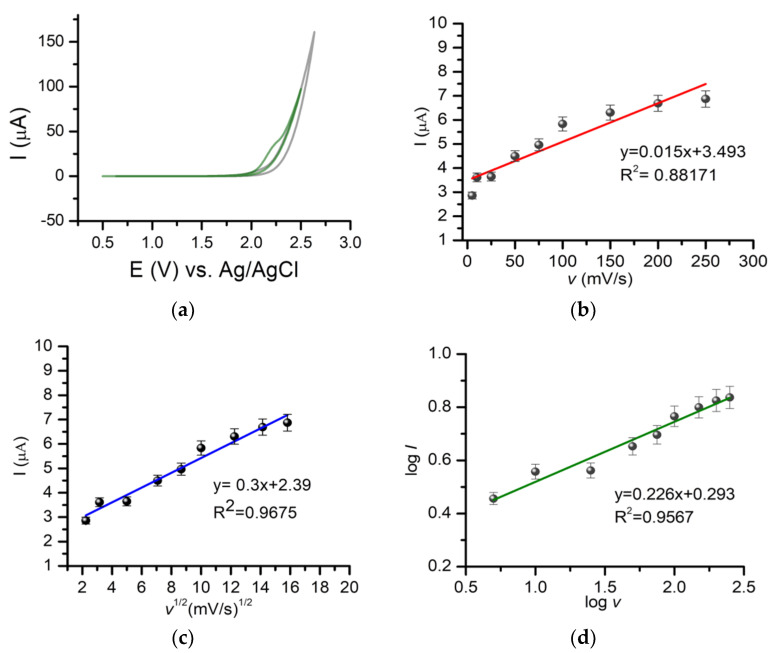
(**a**) CVs obtained for a PBS solution of pH 7.4 (gray) and 25 μg/mL GMB standard solution (green) (**b**) The variation of the oxidation current with the scan rate. (**c**) The variation of the oxidation current with the square root of the scan rate. (**d**) Variation of log(I) with log(v).

A DPV analysis done on BDDE using different scan rates (5 mV/s–200 mV/s) showed, in general, an increase of the oxidation signal with an increase of the scan rate and a shift of the oxidation peak towards positive values of potential. Tests performed using cyclic voltammetry (CV) showed that the oxidation of GMB was irreversible (Figure 2a). The variation of the oxidation current with the square root of the scan rate was also investigated, and a better linearity was observed between the intensity of the current and square root of the scan rate (Figure 2b,c), suggesting that diffusion is the process that controls the electrochemical oxidation of GMB. Representing log(I) depending on log(v), the equation from Figure 2d was obtained, with the slope value of 0.226, closer to 0.5, specific for diffusion-governed electrochemical processes. Similar observations were made in another study found in the literature but using GCE [21]. For further analyses, a scan rate of 100 mV/s was chosen.

### 2.2. Detection of Gemcitabine Using Voltammetry

**Figure 3:**
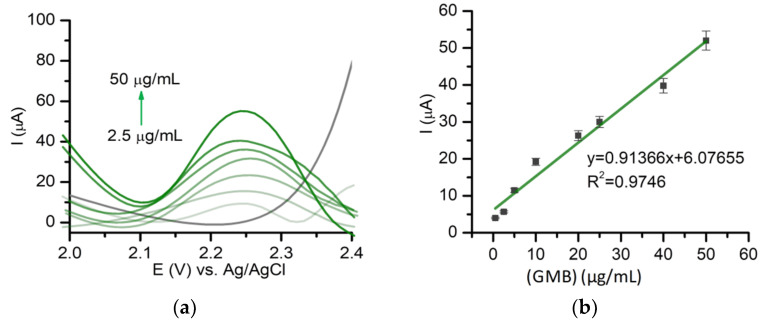
(**a**) DPVs of different concentrations of GMB in PBS (pH 7.4; 0.05 M) (gray—PBS pH 7.4, light green to dark green—2.5–50 µg/mL. (**b**) Calibration curve of GMB in PBS (pH 7.4; 0.05 M) using DPV on BDDE.

Using the optimized DPV procedure, a calibration curve was built using concentrations between 2.5 μg/mL and 50 μg/mL GMB in PBS (pH 7.4; 0.05 M) (Figure 3a,b). The limit of detection (LOD) was calculated based on the signal-to-noise ratio of 3 (S/N = 3), and the value obtained was 0.85 μg/mL, while a limit of quantification (LOQ) of 2.5 μg/mL based on a S/N = 10 was identified. The LOQ was also experimentally tested. The sensitivity of the optimized DPV method (the slope of the calibration curve presented in Figure 3b) was 0.9137 μA mL/μg.

### 2.3. Detection of Gemcitabine Using Amperometry

**Figure 4:**
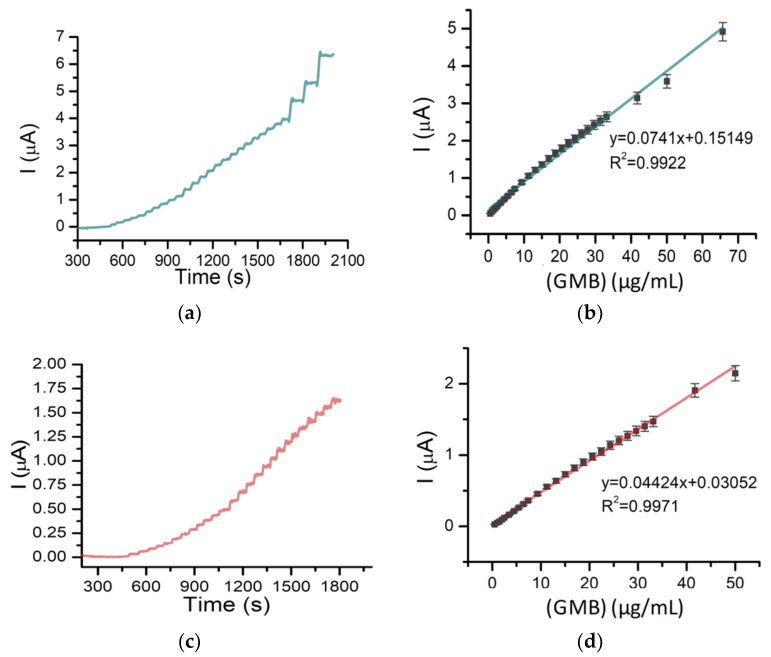
(**a**) Chronoamperogram of successive additions of different volumes of 500 µg/mL GMB solution (5 × 5 µL, 5 × 10 µL, 14 × 20 µL, 2 × 100 µL and 1 × 200 µL) in PBS (pH 7.4; 0.05 M) on BDDE. (**b**) Calibration curve of GMB in PBS (pH 7.4; 0.05 M) using amperometry on BDDE. (**c**) Chronoamperogram of successive additions of different volumes of 500 µg g/mL GMB (5 × 5 µL, 5 × 10 µL and n × 20 µL) in PBS (pH 5.5; 0.05 M) on BDDE. (**d**) Calibration curve of GMB in PBS (pH 5.5; 0.05 M) using amperometry on BDDE.

Based on the results obtained with DPV, amperometry studies were performed for the detection of GMB, and good results regarding the linearity were obtained. The potential of the amperometric procedure was chosen so that the oxidation of GMB would take place and the noise of the baseline would be acceptable in order to detect smaller concentrations compared to the voltammetric method. Therefore, the amperograms were registered at 2.0 V by adding small, measured volumes of a concentrated GMB solution in 5 mL of PBS (pH 7.4; 0.05 M) under continuous stirring (Figure 4a). A calibration curve with good linearity was obtained for GMB concentrations between 0.5 and 65 µg/mL (Figure 4b), thus proving to be a more sensitive detection method than voltammetry. Using the same method, a calibration curve was also built in PBS (pH 5.5; 0.1 M), this pH being often used in antitumor release studies from various drug delivery systems (Figure 4c,d). An LOD of 0.15 μg/mL, an LOQ of 0.5 μg/mL and a sensitivity of 0.074 μA mL/μg were obtained for the optimized amperometric technique.

Despite the lower sensitivity of the electrochemical methods described herein, compared to the methods described in the literature (Table 1), our methods present certain advantages such as ease of use, rapidity, good stability and low cost. Other direct electrochemical methods for the detection of GMB involved the use of surfactants [16], which cannot be used in the case of solutions that need to be administered to patients after analysis or the use of gold electrodes [17], on which, in our work, there was no observed oxidation peak of GMB. Indirect approaches can lower the LOD for GMB, with LODs in the femtomolar range reported in previous works [18]. However, these methods require the functionalization of the electrode surface with molecularly imprinted polymers (MIPs), which can be difficult to obtain and can also pose reproducibility problems. Other works included the use of electrodes modified with MIPs that quantified GMB by measuring its interaction with DNA strands [19,20]. The modified electrodes are also difficult to obtain, and the use of DNA can pose stability issues.

### 2.4. Real Sample Analysis

**Figure 5:**
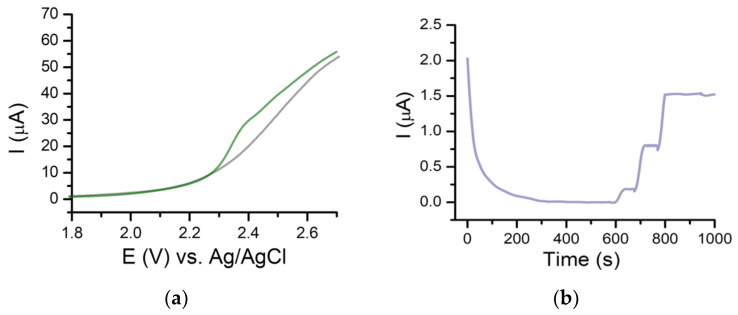
(**a**) DPVs obtained in PBS solution of pH 7.4 (gray) and 10 μg/mL GMB infusion solution (green). (**b**) Chronoamperogram of successive additions of 25, 90 and 140 μL of 500 μg/mL GMB infusion solution in 5 mL PBS (pH 7.4; 0.05 M) corresponding to 2.5, 10 and 25 μg/mL GMB.

In order to check the applicability of the developed method, GMB hydrochloride powder for infusion solution was acquired, and solutions of 2.5, 10 and 25 μg/mL in PBS (pH 7.4; 0.05 M) were prepared and tested with the optimized DPV procedure (Figure 5a). The same concentrations were obtained and tested by amperometry by adding the right volumes (25, 90 and 140 μL, respectively, of pharmaceutical stock solution of 500 μg/mL) in 5 mL of PBS (pH 7.4; 0.05 M) under continuous stirring, registering, in this case, the variation of the intensity of the current in time (Figure 5b). Using the calibration curves built, the concentrations of the solutions were determined, and the recoveries and the relative standard deviations (RSDs) were calculated (Table 2).

Considering the results obtained, a new pharmaceutical formulation—this time, a concentrated solution for infusion—was procured, and dilutions of the same concentrations were prepared and tested on the same day by using the electrochemical methods developed, UV-Vis spectrophotometry and HPLC-UV. The recoveries obtained (Table 3) were statistically compared through ANOVA.

The pharmaceutical formulations tested contained, besides GMB, mannitol, sodium acetate trihydrate, hydrochloric acid and sodium hydroxide for pH adjustments in the case of powder for the infusion solution and macrogol 300, propylene glycol, anhydrous ethanol, hydrochloric acid and sodium hydroxide for the pH adjustment in the case of the concentrated solution for infusion. None of these ingredients had a significant influence on the detection of GMB, since the calculated recoveries were very good.

### 2.5. Robustness of the Applied Electrochemical Methods for Pharmaceutical Samples Analysis

The robustness or the intra-assay variation of the data obtained by using the methodologies applied to determine the GMB content of the pharmaceutical products was tested. The recoveries were calculated for three different concentrations, with three determinations of each concentration. The dataset obtained was statistically analyzed by ANOVA, and the robustness results were good, as can be seen in Table 4.

In all the experiments, regression was highly significant, and no deviation was found in either the parallelism or the linearity (*p* > 0.05). Moreover, all the assays gave results within the confidence interval, which means that the assay system was properly executed. Simultaneously, the developed methods were found to have significant response differentiations between the concentrations and significant sensitivity to the selected concentrations (Table 4).

### 2.6. Correlation and Comparison of Methods

**Figure 6:**
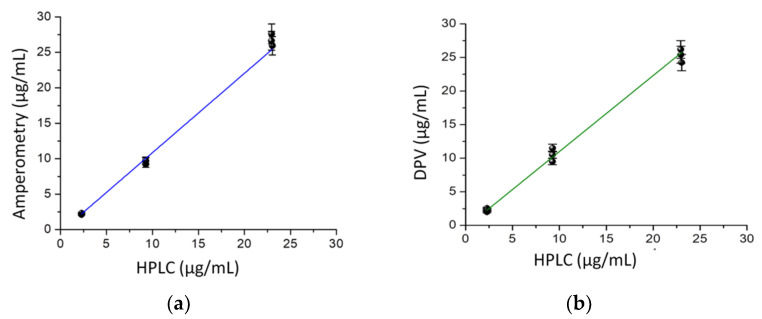
(**a**) Correlation between the determined concentrations of GMB (solutions of different concentrations prepared using a pharmaceutical formulation) obtained by: amperometry, DPV (**b**) and the HPLC-UV control method; the concentration range for GMB: from 2.5 to 25 μg/mL (*n* = 9).

In order to establish a comparison between the proposed methods: amperometry and DPV and the reported HPLC-UV method, we applied these techniques for the analysis of pharmaceutical products containing GMB ranging from 2.5 to 25 μg/mL. A *t*-test (two-sample assuming equal variances) was performed, and the results indicated that there is no significant difference between the series (*p* = 0.11 > 0.05). The correlation between the concentrations of GMB in real samples assessed by the amperometry and DPV assay versus those observed from the HPLC-UV assay was estimated and graphically represented in Figure 6a,b. As can be seen, the results indicated a dose-dependent relationship and insignificant difference between the tested methods, with an acceptable regression coefficient of 0.988 (*p* < 0.001) and a slope of 1.041.

**Figure 7:**
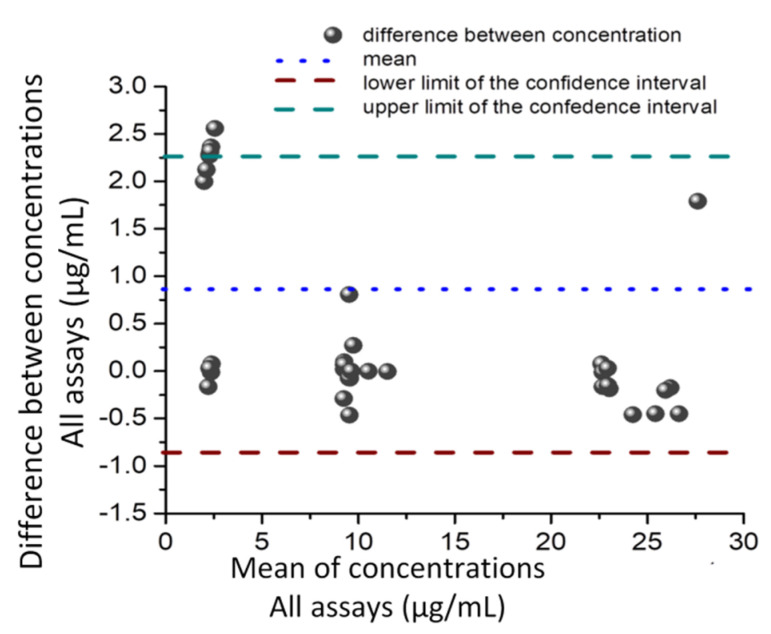
Bland-Altman analysis for GMB determinations in pharmaceutical compounds by amperometry, DPV, UV-VIS and by the HPLC-UV assays. Plotted points represent the differences between concentrations measured by the four methods versus the mean of these methods. The medium dotted horizontal line is the estimated mean bias of 0.725 µg/mL, and the two short, dashed horizontal lines represent the upper (green) and lower (brown) limits of the 95% confidence interval for agreement determined by the mean SD = ±5.31.

The Bland-Altman plot (Figure 7) was used to measure the agreement of the results obtained from the GMB amperometric, voltammetric, UV-VIS and the HPLC-UV assays. This plot illustrates the differences between all the datasets versus the mean of the GMB concentrations recorded using the above-mentioned methods. The mean difference in concentrations gained by the four procedures was 0.713, with limits of agreement of −0.805 and +2.233 (Figure 7). This shows a strong agreement between the four methods applied for determining the GMB in the pharmaceutical samples, with 95% of the differences lying between the limits. Based on the Bland-Altman plot, it can be concluded that the four analytical methods are in good correspondence with each other and that the accuracy and robustness of the optimized electrochemical detection strategies are very good.

## 3. Materials and Methods

### 3.1. Materials and Equipment

#### 3.1.1. Chemicals and Reagents

For the preparation of GMB standard solutions, gemcitabine hydrochloride > 98% bought from TCI (Tokyo Chemical Industry), Tokyo, Japan was used. Phosphoric acid (H_3_PO_4_), potassium dihydrogen phosphate (KH_2_PO_4_), disodium hydrogen phosphate (Na_2_HPO_4_) sodium chloride (NaCl), acetic acid (CH_3_COOH), sodium acetate (CH_3_COONa), boric acid (H_3_BO_3_), sodium hydroxide (NaOH), sulphuric acid (H_2_SO_4_), citric acid, sodium citrate, hydrochloric acid (HCl), sodium carbonate (Na_2_CO_3_), sodium bicarbonate (NaHCO_3_), chloroauric acid (HAuCl_4_) and methanol were purchased from Sigma Aldrich, St. Louis, MO, USA. All reagents were of analytical grade and were used without further purification. All solutions were prepared using Milli-Q ultrapure water (18.2 MΩ, Millipore Simplicity, Sigma Aldrich, St. Louis, MO, USA).

Gemcitabine powder for the infusion solution (Gemcirena^®^ 38 mg/mL, Fresenius Kabi, Bad Homburg, Germany) and gemcitabine concentrate for the infusion solution (Gemcitabina Accord^®^ 1000 mg, Accord Healthcare, Warsaw, Poland) were used for the pharmaceutical sample analysis.

#### 3.1.2. Equipment

A multichannel potentiostat/galvanostat Autolab MAC80100 (Metrohm, Utrecht, The Netherlands) operated with Nova 1.10.4 software was used to perform all the electrochemical tests. Different working electrodes were used: graphite, gold and platinum-based screen-printed electrodes (SPE, 4-mm diameter) with a silver pseudo-reference and a carbon counter-electrode (provided by Metrohm DropSens, Madrid, Spain), glassy carbon electrode (GCE, 3-mm diameter) (bought from BASi, West Lafayette, IN, USA), a boron-doped diamond electrode (BDDE, 3-mm diameter) (produced by Windsor Scientific, Berkshire, UK) and pencil graphite electrodes (PGE, 1-mm diameter) of HB (hard black) hardness bought from Rotring. All experiments using the BDDE, GCE and PGE were performed using a conventional three-electrode cell using a solid Ag/AgCl (eDAQ Pty Ltd., Denistone East, Australia) reference electrode and a platinum wire as the counter-electrode. The SPEs were used as received, while, for the other electrodes, a cleaning procedure was used before each test, as follows: the BDDE was polished with 3-μm diamond polish and sonicated for 30 min in ultrapure water. The GCE was polished with 3-μm alumina slurry and sonicated for 2 min in each of the following solvents: acetone, isopropanol, ethanol and ultrapure water. The cleaning of the PGE was performed by immersing the electrode in acetone for 1 min, followed by an electrochemical cleaning process in 0.5-M H_2_SO_4_ using CV between −1 V and 1 V with a scan rate of 100 mV/s for 5 cycles.

The pH of the solutions was measured using a pH meter HI208 (Hanna Instruments, Smithfield, RI, USA).

For the spectrophotometric analyses, a SPECORD 250 PLUS UV-VIS spectrophotometer (Analytik Jena AG, Jena, Germany) was used.

An HPLC analysis was performed using an Agilent 1100 Series HPLC system (Agilent Technologies, Santa Clara, CA, USA) equipped with an ultraviolet (UV) detector and a Zorbax C18 column (100 × 3 mm, internal diameter 3.5 μm) (Agilent Technologies, Santa Clara, CA, USA).

### 3.2. Methods

#### 3.2.1. Buffer Preparation

BRB solutions of pH from 2 to 12 were prepared by preparing a solution of 40 mM phosphoric acid, acetic acid and boric acid and adjusting the pH to the desired value using a concentrated sodium hydroxide solution.

PB solutions were prepared according to the European Pharmacopoeia. For PB (pH 5.5; 0,1 M), 13.61 g potassium dihydrogen phosphate and 35.81-g disodium hydrogen phosphate were dissolved each in 1000 mL ultrapure water. After that, 96.4 mL of the first solution were mixed with 3.6 mL of the second. For PB (pH 7.4; 0.05 M), a 50 mL solution of 0.2-M potassium dihydrogen phosphate was mixed with 39.1 mL of 0.2 M sodium hydroxide solution, and ultrapure water was added to 200 mL. In order to obtain PBS (pH 5.5; 0.1 M) and PBS (pH 7.4; 0.05 M), 0.85 g of sodium chloride were added to 100 mL of the corresponding PB solution.

The AB solution (pH 4.5; 0.1 M) was prepared by dissolving 0.369 g sodium acetate in ultrapure water, adding 0.33 g acetic acid and adjusting the volume to 100 mL. A citrate solution containing 2.427 g of sodium citrate dihydrate and 0.336 g of citric acid dissolved in 100 mL was prepared in order to obtain the CB solution (pH 6; 0.1 M). The CBB solution (pH 10; 0.1 M) was prepared by dissolving 0.388 g sodium bicarbonate and 0.571 g sodium carbonate in ultrapure water, and the volume was adjusted to 100 mL.

In all cases, the pH of the solutions was registered and adjusted if necessary using concentrated hydrochloric acid or sodium hydroxide solutions.

#### 3.2.2. Voltammetry Study

A redox process was followed for GMB using the above-mentioned electrode materials in CV in a potential range between 0 and 1.5 V at 100 mV/s for the SPEs, PGE and GCE. PGEs functionalized with AuNPs in a 2.5 mM chloroauric acid solution in 0.5 M sulphuric acid via CV were also tested in the same conditions. The oxidation of GMB on BDDE was followed in CV in a potential range between 0.5 and 2.5 V at 100 mV/s.

The influence of the pH on the detection of GMB was evaluated in BRB solutions of pH 2–12. Solutions of GMB (100 μg/mL) in 2 mL of 0.1-M sulphuric acid and all buffer solutions mentioned in Section 3.2.1 were prepared and tested using DPV. The DPV parameters were experimentally optimized to allow the highest current signal for the electrochemical oxidation of gemcitabine. The optimized values were as follows: a step potential of 0.01 V, modulation amplitude 0.05 V, modulation time 0.02 s, interval time 0.1 s and scan rate 100 mV/s.

The variation of the analytical signal with the scan rate was analyzed in DPV from 5 mV/s to 200 mV/s using 2 mL of 100 μg/mL GMB solution in PBS (pH 7.4; 0.05 M).

After the optimization of the electrochemical method, a calibration curve was built for GMB in PBS (pH 7.4; 0.05 M) using DPV (concentration range: 0.5–50 μg/mL). The LOD was calculated based on the S/N = 3, while the LOQ was estimated based on the S/N = 10 and experimentally tested. The sensitivity of the method was determined as the slope of the calibration curve.

#### 3.2.3. Amperometry Study

GMB was also determined using an amperometry procedure at 1.9 V by following the current leap when specific volumes of GMB 500 μg/mL solution were added in 5 mL PBS under continuous stirring. A calibration curve for GMB was built with this technique in PBS (pH 7.4; 0.05 M), as well as in PBS (pH 5.5; 0.1 M). The LOD, LOQ and sensitivity were estimated in the same way as for the DPV method.

#### 3.2.4. UV-Vis Spectrophotometry

The UV-Vis spectrum of a standard GMB solution (10 μg/mL in PBS pH 7.4; 0.05 M) was recorded in order to find the maximum of absorption. Then, standard solutions of GMB (2.5, 10 and 25 μg/mL in PBS (pH 7.4; 0.05 M)), as well as GMB pharmaceutical formulations (2.5, 10 and 25 μg/mL in PBS (pH 7.4; 0.05 M)) were analyzed in the same conditions at the maximum of absorption (λ_max_ = 270 nm).

#### 3.2.5. HPLC-UV Analysis

The HPLC analysis was performed using as the mobile phase phosphoric acid 0.1% (*v*/*v*)–methanol (97:3, *v*/*v*), with a flow rate of 0.5 mL/min and UV detection at 272 nm. The injection volume of the samples was 50 μL, the analysis time was 5 min and the retention time (t_r_) registered for GMB was 1.6 min. The samples were prepared in PBS (pH 7.4; 0.05 M). A calibration curve for GMB, in the concentration range of 0.5–12 µg/mL (y = 250.41x + 7.239), was built.

#### 3.2.6. Real Sample Analysis

A solution of an equivalent of 2 mg/mL GMB was prepared from each pharmaceutical formulation mentioned in Section 3.1.1, and dilutions of 2.5, 10 and 25 μg/mL were prepared and tested.

The pharmaceutical formulation solutions were prepared as follows: in order to obtain the abovementioned concentrations, the necessary amount of powder for the infusion solution was calculated and accurately weighed in a 2 mL flask and then dissolved in PBS (pH 7.4; 0.05 M) to obtain 2 mL of solution. For the concentrate for the infusion solution, the necessary volume was calculated and measured using a micropipette and then diluted to 2 mL with PBS (pH 7.4; 0.05 M) to obtain the desired concentrations.

The samples originating from the second pharmaceutical formulation were tested using electrochemical techniques (DPV and amperometry), as well as UV-Vis spectrophotometry and HPLC-UV, and the correlation between these methods was evaluated. The same preparation methodology was used for the samples analyzed by all the methods.

#### 3.2.7. Robustness of the Applied Electrochemical Methods for Pharmaceutical Sample Analysis

The recoveries calculated for the target analyte using four different methods: two electrochemical, HPLC-UV and UV-VIS spectroscopy were evaluated comparatively in terms of robustness between methods. Robustness of the applied electrochemical methods for the pharmaceutical samples analysis was evaluated by comparing the electrochemical data with the ones obtained using the HPLC-UV method, the analytical technique recommended by the European Pharmacopoeia. The variation (intra-assay) of the recoveries between the above-mentioned assays was statistically analyzed by the ANOVA protocol (Microsoft Office Excel 2010 software option).

#### 3.2.8. Correlation and Comparison of the Methods

The results obtained in this study with the amperometric, as well as with the DPV-based detection procedure, were compared against the ones obtained with a characterized HPLC-UV procedure. The precision results of the methods were statistically analyzed using the *t*-test (two-sample assuming equal variances), which indicates whether there is a significant difference between the methods at a 5% significance level.

## 4. Conclusions

A simple, fast and direct electrochemical method based on the oxidation of GMB at BDDE was successfully developed and applied for the determination of the above-mentioned drug in real samples. The electrochemical behavior of GMB was investigated using CV and DPV, with several electrodes and experimental conditions being tested. The oxidation process was irreversible, with an anodic oxidation peak at around 2.2 V on BDDE in PBS (pH 7.4; 0.05 M).

The optimized DPV method demonstrated high precision and accuracy at concentrations ranging from 2.5 to 50 μg/mL. Based on these results, an amperometric method was also optimized and showed increased sensitivity and linearity in the range of 0.5–65 μg/mL. These methods were both applied to the pharmaceutical formulations, with good recoveries and no significant interferences from the pharmaceutical formulations’ components.

The amperometric and voltametric detection strategies present several advantages, including their simplicity, short analysis time (30 s using DPV and 300 s using amperometry) and low cost, becoming increasingly appropriate when an HPLC-UV system is not available for determining the content of the drug in real samples. The methods use simple reagents, require minimum sample preparation procedures and generate no toxic residues, encouraging their application in a routine analysis. These two detection strategies were optimized for physiological pH, thus demonstrating that they could be potentially used in the future for the pharmaceutical formulation quality control in hospitals. The results obtained using DPV and amperometry were compared to UV-Vis and HPLC-UV as the control methods, and the statistical analysis demonstrated good correspondence between the methods, as well as a high accuracy and robustness. Based on this finding, it could be concluded that there was no statistical difference between the reported assays and the control HPLC-UV and UV-Vis methods for GMB quantification. Therefore, these methods can be interchangeable. The results proved that electrochemical assays are excellent alternative methods for analyzing GMB in medicines, being useful tools to supplement or replace conventional physiochemical methods for biomedical applications.

## Figures and Tables

**Table 1:** Comparison of the analytical parameters of different GMB detection methods reported in the literature.

Electrode	Detection Method	Linear Range (μg/mL)	LOD (μg/mL)	Ref
CPE, bare + surfactant	DPV	0.140–89.900	0.0026	16
AuE, bare	DPV	0.026–3.947	0.0160	17
AuE/MMOF	LSV	1.1 × 10^−9^–0.011	9 × 10^−10^	18
GCE/MIP/dsDNA	DPV	1.000–30.000	0.2760	19
CPE/MIP/MWCNT/AgNP/dsDNA	DPV	0.394–24.470	0.0033	20
BDDE	DPV	2.500–50.000	0.8500	This work
BDDE	AMP	0.500–65.000	0.1500	This work

CPE—carbon paste electrode, AuE—gold electrode, MMOF—microporous metal organic framework, MWCNT/AgNP/dsDNA—multiwalled carbon nanotubes/silver nanoparticles/double-stranded DNA, AMP—amperometry and LSV—linear sweep voltammetry.

**Table 2:** Results obtained with the electrochemical methods optimized for solutions prepared from GMB powder for the infusion solution.

(GMB) (μg/mL)	Method	Recovery (%)	RSD (%)
2.5	DPV	93.2	0.40
10		100.6	9.98
25		96.88	6.30
2.5	Amperometry	83.33	1.26
10		82.35	7.41
25		93.39	8.53

**Table 3:** Results obtained with the electrochemical methods optimized, UV-Vis spectrophotometry and HPLC-UV for solutions prepared from the GMB-concentrated solution for infusion.

(GMB) (μg/mL)	Method	Recovery (%)	RSD (%)
2.5	DPV	92.22	12.35
10		105.12	9.48
25		101.14	3.86
2.5	Amperometry	89.47	4.54
10		95.40	2.68
25		106.91	3.15
2.5	UV-Vis	92.67	0.08
10		95.42	0.33
25		90.65	0.83
2.5	HPLC-UV	91.33	0.15
10		92.61	0.06
25		91.90	0.23

**Table 4:** Statistical results obtained using ANOVA for recovery values calculated with the four methods.

Source of Variation	SS	df	MS	F	*p*-Value	F Crit
Between Groups	344.95	3	114.98	2.71	0.06114	2.90
Within Groups	1355.54	32	42.36			
					*p*-value theoretical	
Total	1700.49	35			0.05	

## Data Availability

The experimental data are stored in the authors’ laboratory and can be consulted after a prior request to the corresponding author.

## Supplementary Materials

The following are available online at https://www.mdpi.com/article/10.3390/ph14090912/s1: Figure S1: The voltammograms registered using the optimized DPV procedure for phosphate buffer saline (PBS) solution of pH 7.4 (black) and 20 μg/mL gemcitabine (GMB) in PBS (pH 7.4; 0.05 M). Figure S2: The variation of the oxidation potential of gemcitabine (GMB) with the pH of the electrolyte solution.
