# Supplementary material for: Gemcitabine Direct Electrochemical Detection from Pharmaceutical Formulations Using a Boron-Doped Diamond Electrode
Source: Pharmaceuticals (Basel). 2021 Sep 10;14(9):912. doi: 10.3390/ph14090912 (PMC8466666; doi:10.3390/ph14090912)
Supplement: Supplementary file 1 [file pharmaceuticals-14-00912-s001.zip › pharmaceuticals-1348570-supplementary.pdf]

Gemcitabine Direct Electrochemical Detection from Pharmaceutical Formulations Using Boron Doped Diamond Electrode

Iulia Rus [1#], Alexandra Pusta [1,2#], Mihaela Tertiș [1], Cristina Barbălată [3], Ioan Tomuță [3], Robert Săndulescu [1] and Cecilia Cristea [1,*]

[1] Department of Analytical Chemistry, Iuliu Hațieganu University of Medicine and Pharmacy, 4 Louis Pasteur Street 400349, Cluj-Napoca, Romania; rus.iulia@umfcluj.ro (I.R.); alexandrapusta@gmail.com (A.P.); mihaela.tertis@umfcluj.ro (M.T.); rsandulescu@umfcluj.ro (R.S.); ccristea@umfcluj.ro (C.C.)

[2] Department of Medical Devices, Iuliu Hațieganu University of Medicine and Pharmacy, 4 Louis Pasteur Street 400349, Cluj-Napoca, Romania; alexandrapusta@gmail.com

[3] Department of Pharmaceutical Technology and Biopharmaceutics, Iuliu Hațieganu University of Medicine and Pharmacy, 41 Victor Babeș Street 400012, Cluj-Napoca, Romania; barbalata.cristina@umfcluj.ro (C.B.); tomutaioan@umfcluj.ro (I.T.)

* Correspondence: ccristea@umfcluj.ro; 4 Louis Pasteur Street 400349, Cluj-Napoca, Romania; Tel.: +40 721 375 789

[#] Authors with equal contributions

S1: The DPVs registered for 20 µg/mL gemcitabine in the range of 0.5-2.5 V on BDDE compared with the one registered in the same potential range in the absence of the analyte

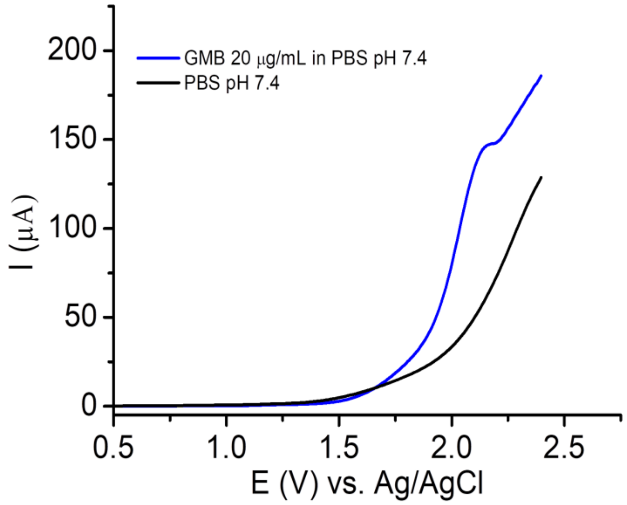

Figure S1. The voltammograms registered using the optimized DPV procedure for phosphate buffer saline (PBS) solution of pH 7.4 (black) and 20 µg/mL gemcitabine (GMB) in PBS (pH 7.4; 0.05 M)

S2: The dependence between the oxidation potential of gemcitabine and the pH of the electrolyte

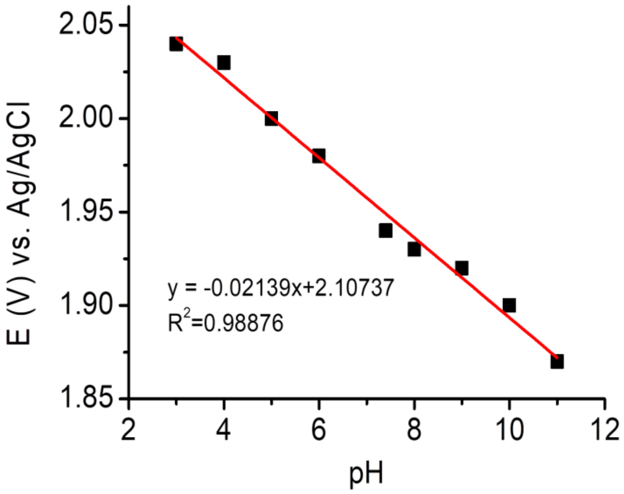

Figure S2. The variation of the oxidation potential of gemcitabine (GMB) with the pH of the electrolyte solution.
